# Supplementary material for: Predicting visual function by interpreting a neuronal wiring diagram
Source: Nature. 2024 Oct 2;634(8032):113–23. doi: 10.1038/s41586-024-07953-5 (PMC11446822; doi:10.1038/s41586-024-07953-5)
Supplement: Supplementary file 6 — CRF and ERF predictions for individual TmY4 and TmY9 cells. Analogous to Supplementary Data 3, but for TmY target types. Shown are the top four monosynaptic pathways, the strongest pathway passing through each of the top ten intermediary types (ranking from Extended Data Fig. 7), and the trisynaptic pathway Tm1–TmY–Dm3–TmY (see the section entitled Prediction of spatial normalization). [file 41586_2024_7953_MOESM6_ESM.zip › DataS4/TmY9q/720575940607726857.pdf]

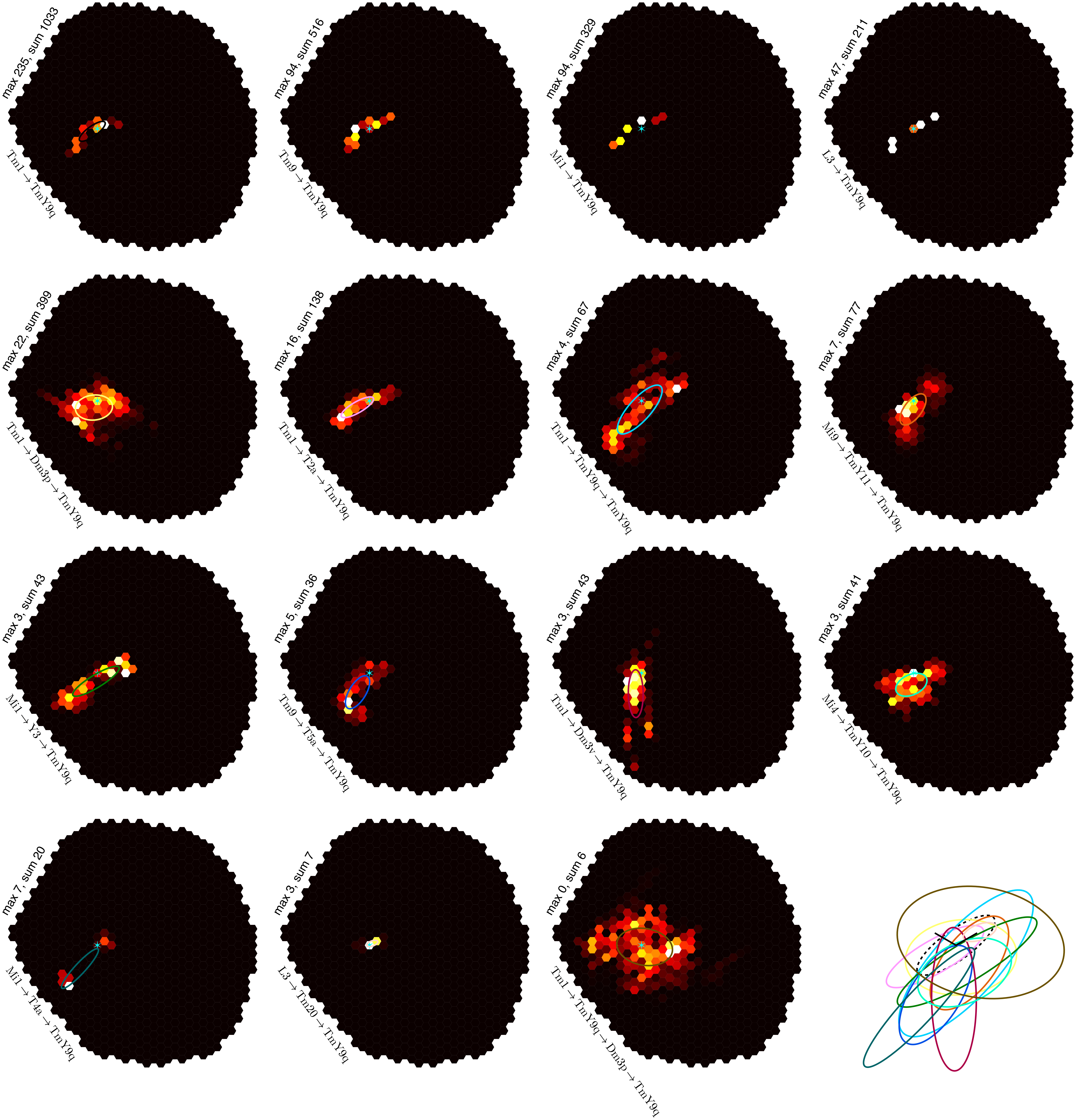
max 235, sum 1033
Tm1 → TmY9q
max 94, sum 516
Tm9 → TmY9q
max 94, sum 329
Mi1 → TmY9q
max 47, sum 211
L3 → TmY9q
max 22, sum 399
Tm1 → Dm3p → TmY9q
max 16, sum 138
Tm1 → T2a → TmY9q
max 4, sum 67
Tm1 → TmY9q → TmY9q
max 7, sum 77
Mi9 → TmY11 → TmY9q
max 3, sum 43
Mi1 → Y3 → TmY9q
max 5, sum 36
Tm9 → T5a → TmY9q
max 3, sum 43
Tm1 → Dm3v → TmY9q
max 3, sum 41
Mi4 → TmY10 → TmY9q
max 7, sum 20
Mi1 → T4a → TmY9q
max 3, sum 7
L3 → Tm20 → TmY9q
max 0, sum 6
Tm1 → TmY9q → Dm3p → TmY9q
